# Preparation of TiO_2 _nanotube/nanoparticle composite particles and their applications in dye-sensitized solar cells

**DOI:** 10.1186/1556-276X-7-48

**Published:** 2012-01-05

**Authors:** Chang Hyo Lee, Seung Woo Rhee, Hyung Wook Choi

**Affiliations:** 1 Department of Electrical Engineering, Kyungwon University, San 65 Bokjeong-dong, Sujeong-gu, Seongnam, Gyeonggi-do, 461-701, Seoul, Korea

**Keywords:** composites, chemical synthesis, X-ray diffraction, electron microscopy, optical properties.

## Abstract

Efficiency of dye-sensitized solar cells [DSSCs] was enhanced by combining the use of TiO_2 _nanotubes [TNTs] and nanoparticles. TNTs were fabricated by a sol-gel method, and TiO_2 _powders were produced through an alkali hydrothermal transformation. DSSCs were constructed using TNTs and TiO_2 _nanoparticles at various weight percentages. TNTs and TiO_2 _nanoparticles were coated onto FTO glass by the screen printing method. The DSSCs were fabricated using ruthenium(II) (N-719) and electrolyte (I_3_/I_3_^-^) dyes. The crystalline structure and morphology were characterized by X-ray diffraction and using a scanning electron microscope. The absorption spectra were measured using an UV-Vis spectrometer. The incident photocurrent conversion efficiency was measured using a solar simulator (100 mW/cm^2^). The DSSCs based on TNT/TiO_2 _nanoparticle hybrids showed better photovoltaic performance than cells made purely of TiO_2 _nanoparticles.

## Introduction

Dye-sensitized solar cells [DSSCs] have been intensively studied following their discovery in 1991. DSSCs have been extensively researched over the past decades due to their high energy-conversion efficiency and especially their low production cost as cheaper alternatives to silicon solar cells [1-3]. A DSSC is composed of a dye-adsorbed nanoporous TiO_2 _layer on a fluorine-doped tin oxide [FTO] glass substrate, redox electrolytes, and a counter electrode. A unidirectional charge flow with no electron leakage at the interfaces is essential for high energy-conversion efficiency [4]. The energy-conversion efficiency is likely to be dependent on the morphology and structure of the dye-adsorbed TiO_2 _film. Ito et al. introduced mesoporous TiO_2 _particular films as photoanodes to enhance the effective surface area, to absorb more dye molecules, and thus, to achieve more light absorption and greater efficiency [5,6]. The high conversion efficiency achieved by the DSSC may be attributed to its uniquely porous titania film, which is usually prepared with titania nanoparticles. Sol-gel processing of titanium dioxide has been extensively investigated, and modern processes have been developed to refine and control the stability as well as the phase formation of the colloidal precursors [7]. However, because the mesoporous TiO_2 _particles are randomly connected, this will unavoidably lead to the recombination of electron-hole pairs, decreasing efficiency. Subsequently, researchers started to explore the use of ordinal TiO_2 _in DSSCs; this includes TiO_2 _nanowires, nanorods, and TiO_2 _nanotubes [TNTs]. The preparation of TNTs by a hydrothermal treatment of TiO_2 _powder in a 10-M NaOH aqueous solution has been reported [8,9]. The use of oxide semiconductors in the form of nanorods, nanowires, and nanotubes may be an interesting approach to improve electron transport through the film. Because of the one-dimensional nature of these nanostructures, their morphology facilitates electron transfer up to the collecting electrode, decreasing the ohmic loss through the TNTs [10-13]. To improve electron transport, provide a large surface area to adsorb the sensitized dye, and enhance incident light harvest, the use of TNTs in DSSCs has been explored [14,15]. In the present work, the effect of combining TiO_2 _nanoparticles with TNTs and the resulting effect on solar cell performance have been investigated. DSSCs were constructed by the application of TNTs and TiO_2 _nanoparticles at various weight ratios. TNTs were fabricated by a hydrothermal-temperature process using the sol-gel method. TiO_2 _powder was produced through alkali hydrothermal transformation. The introduction of TNTs, with a much more open structure, enables the electrolyte to penetrate easily inside the film, increasing the interfacial contact between the nanotubes, the dye, and the electrolyte. In addition, a high level of dye adsorption on TiO_2 _in the form of nanorods and nanotubes is expected because of the high surface area of these nanostructures. It is expected that the photoelectrical performance of the DSSC can be further improved.

## Experimental details

### Preparation of TiO_2 _nanoparticles and nanotubes

The TiO_2 _main layer was prepared using the sol-gel method. Nano-TiO_2 _was synthesized using titanium(IV) isopropoxide [TTIP] (Aldrich Chemical, Sigma-Aldrich Corporation, St. Louis, MO, USA), nitric acid, ethyl alcohol, and distilled water. The TTIP was mixed with ethanol, and distilled water was added drop by drop under vigorous stirring for 1 h. This solution was then peptized using nitric acid and heated under reflux at 80°C for 8 h. After this period, a TiO_2 _sol was prepared. The prepared sol was dried to yield a TiO_2 _powder. The TiO_2 _particles were calcined in air at 450°C for 1 h using a programmable furnace to obtain the desired TiO_2 _stoichiometry and crystallinity. TNTs were prepared using a hydrothermal process described in the authors' previous work. Then, 5 g of TiO_2 _particles prepared by the sol-gel method were mixed with 500 ml of a 10-M NaOH aqueous solution, followed by hydrothermal treatment at 150°C (TNTs) in a Teflon-lined autoclave for 12 h. After the hydrothermal reaction, the treated powders were washed thoroughly with distilled water and 0.1 M HCl and subsequently filtered and dried at 80°C for 1 day. To achieve the desired TNT size and crystallinity, the powders were calcined in air at 500°C for 1 h [16].

### Preparation of TiO_2 _electrode films

TiO_2 _nanoparticles and TNTs prepared by the sol-gel and hydrothermal methods were mixed at various weight ratios (without TNT, 9:1 (10 wt.%), 8:2 (20 wt.%), 7:3 (30 wt.%), 5:5 (50 wt.%), and 100 wt.% TNTs; total weight 6 g) and ground in a mortar. Acetic acid (1 ml), distilled water (5 ml), and ethanol (30 ml) were added gradually drop by drop to disperse the TiO_2 _nanoparticles and nanotubes under continuous grinding. The TiO_2 _dispersions in the mortar were transferred with an excess of ethanol (100 ml) to a tall beaker and stirred with a 4-cm-long magnet tip at 300 rpm. Anhydrous terpineol (20 g) and ethyl celluloses (3 g) in ethanol were added, followed by further stirring. The dispersed contents were concentrated by evaporating the ethanol in a rotary evaporator. The pastes were finished by grinding in a three-roller mill [17]. An optically transparent conducting glass (FTO, sheet resistance 8 Ω/sq) was washed in ethanol and deionized water in an ultrasonic bath for 10 min. The FTO glass was immersed in a 40-mm-deep TiCl_4 _aqueous solution at 70°C for 30 min to make good mechanical contact. A TiO_2 _film with a thickness of 12 to 15 μm was deposited onto the pretreated conducting glass using the screen printing technique and sintered again at 450°C for 15 min and at 500°C for 15 min in air.

### Assembly of the DSSCs

The nanoporous TiO_2 _electrode films were immersed in the dye (N-719) complex for 24 h at room temperature. A counter electrode was prepared by spin-coating an H_2_PTCl_6 _solution onto the FTO glass and heating at 450°C for 30 min. The dye-adsorbed TiO_2 _electrode and the PT counter electrode were assembled into a sandwich-type cell and sealed with a hot-melt sealant of 50-μm thick. An electrolyte solution was introduced through a drilled hole in the counter electrode. The hole was then sealed using a cover glass.

### Measurements

The phase of the particles obtained at various hydrothermal temperatures was examined by X-ray diffraction [XRD] using a D/MAX-2200 diffractometer with CuKα radiation (Rigaku Corporation, Shibuya-ku, Tokyo, Japan). The morphology and thickness of the prepared TNT layers were investigated by field-emission scanning electron microscopy [FE-SEM] (model S-4700, Hitachi, Chiyoda-ku, Tokyo, Japan). The absorption spectra of the TiO_2 _electrode films were measured using a UV-Vis spectrometer (UV-Vis 8453, Agilent Technologies Inc., Santa Clara, CA, USA). The conversion efficiency of the fabricated DSSC was measured using an I-V solar simulator (McScience, Suwon-si, South Korea). The incident photocurrent conversion efficiency was measured using an IPCE Model Qex7 (PV Measurements, Inc., Boulder, CO, USA). The active area of the resulting cell exposed to light was approximately 0.25 cm^2 ^(0.5 cm × 0.5 cm).

### Results and discussions

### Morphological characterization of TiO_2 _film

**Figure 1:**
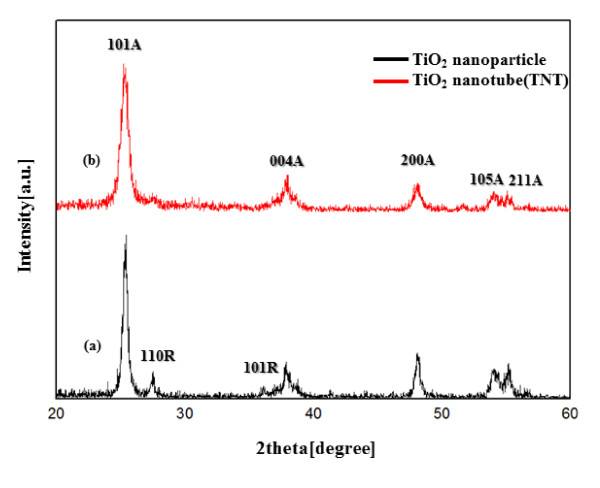
**XRD patterns of TiO_2 _nanoparticles and TNTs**.

**Figure 2:**
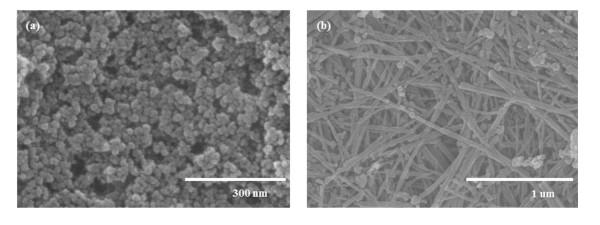
**FE-SEM images of TiO_2 _nanoparticles and TNT films**. (**a**) TiO_2 _nanoparticles made by the sol-gel method and (**b**) TNT films made by the hydrothermal method at 150°C for 12 h.

**Figure 3:**
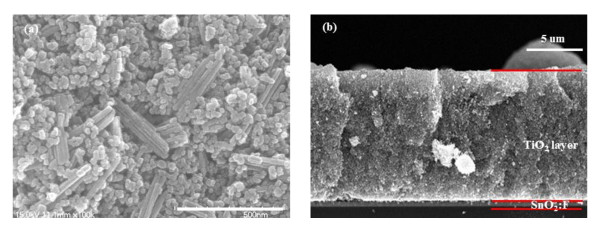
**Cross-sectional (a) and top-view (b) FE-SEM images of the TiO_2 _nanoparticle/TNT composite layer**.

Figure 1a shows the XRD pattern of the sol-gel TiO_2 _nanoparticles at 450°C, which indicates a mixture of the anatase and rutile phases. The XRD pattern of TiO_2 _nanoparticles shows prominent anatase peaks at (101), (004), (200) and prominent rutile peaks at (110) and (101). Figure 1b shows the XRD patterns of the TNT films prepared at hydrothermal temperatures at 150°C for 12 h. The TiO_2 _nanoparticles were observed to be transformed into the anatase phase by the hydrothermal method. As can be observed from the corresponding XRD patterns (Figure 1b), the TNTs possess a highly crystallized anatase structure without any impurity phase. In the TNTs, the rutile peaks indicate that the transformation to anatase is complete. FE-SEM images of the TiO_2 _sol-gel nanoparticles and the TNTs prepared at hydrothermal temperatures are shown in Figure 2a. The diameter of the TiO_2 _nanoparticles prepared by the sol-gel method is consistently about 25 nm. Figure 2b shows an FE-SEM image of the sample anatase TNTs which were grown at 150°C for 12 h and exhibit a pure tube-like structure. The length of the TNTs is several macrometers, their diameter is approximately 50 to 100 nm, and they are very uniform, quite clean, and smooth-surfaced. Figure 3 shows the surface morphology of the electrode film on the FTO glass. Figure 3a shows a film made from TiO_2 _nanoparticles and TNT hybrids, which has a porous structure. A cross-sectional SEM image of the TiO_2 _electrode film (Figure 3b) was also captured. The top part is the TiO_2 _electrode film. The middle one is the FTO layer, and the lowest one is the glass substrate. The electrode is 12- to 15-μm thick in Figure 3b.

### Influence of TNTs on dye adsorption

**Figure 4:**
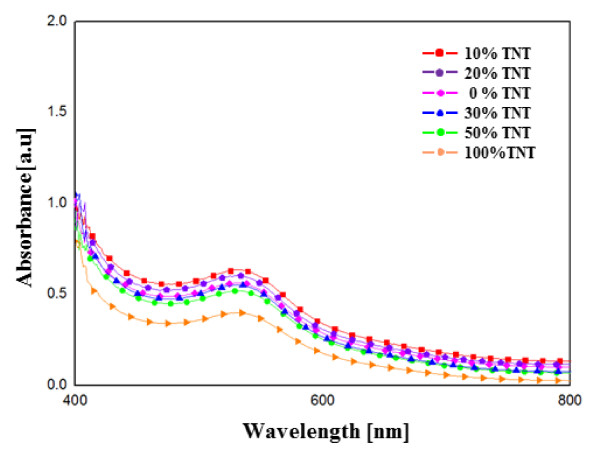
**Absorption spectra of dye from TiO_2 _films containing different ratios of TNTs**.

Figure 4 shows how the UV-Vis absorbance of the TNTs affects the dye-adsorbed TiO_2 _films. It is known that the N-719 dye shows absorption peaks. Figure 4 shows the absorption spectrum of the N-719 dye in the 400- to 800-nm wavelength range in the flexible TiO_2 _electrode film contained with various percentages of TNTs. The TNT content in the TiO_2 _nanoparticles was 0, 10, 20, 30, 50, and 100 wt.%. It can be seen in Figure 4 that in the 400- to 500-nm wavelength range, the absorbance for the sample containing 10 wt.% TiO_2_/TNT was the highest, and the absorbance of the sample containing 100 wt.% TNT was the lowest. The absorption of the nanoparticle film made purely of TiO_2 _was slightly reduced in this region compared to the 10 wt.% and 20 wt.% TNT films. According to Lambert-Beer's law, higher absorbance means a higher dye concentration; a suitable amount of TNT in the film could provide a large surface area for dye adsorption. Therefore, the TiO_2 _layer with the dye serves as the photoactive layer. It is well known that the photocurrent of a flexible DSSC is correlated directly with the number of dye molecules; the more dye molecules are adsorbed, the more incident light is harvested, and the larger is the photocurrent.

### IPCE measurements

**Figure 5:**
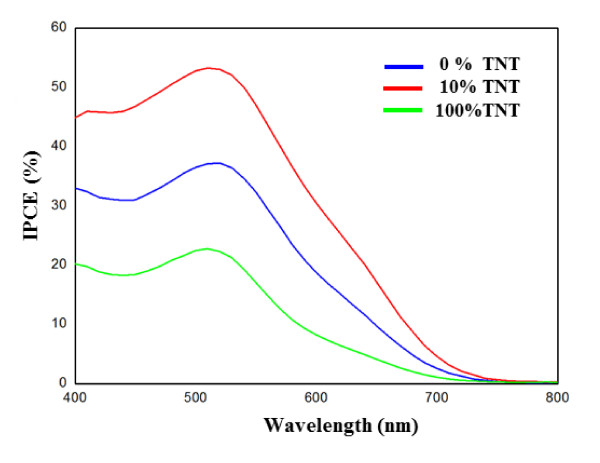
**IPCE action spectra of solar cells**. IPCE action spectra of solar cells made from TiO_2 _films (10 wt.% TNT, 100 wt.% TNT, and without TNT).

The incident photocurrent conversion efficiency [IPCE] is defined as the number of electrons in the external circuit produced by an incident photon at a given wavelength divided by the number of incident photons [18]. The IPCE spectra as a function of wavelength for the TiO_2 _electrode films (10 wt.% TNT, 100 wt.% TNT, and without TNT) are shown in Figure 5. The maximum efficiency at the 510-nm wavelength coincides with the maximum absorption wavelength of the N-719 dye. The IPCE peak height at 510 nm for the 10 wt.% TiO_2_/TNT cell is 53.3%, which is much higher than the values of 15.1% obtained for the 100 wt.% TNT cell and 37.2% for the cell without TNT. Furthermore, over the whole spectral region, the 10 wt.% TNT cell exhibits considerable higher IPCE values than the other two samples. Based on the experimental results and data analysis described above, the constructed TiO_2_/TNT (10 wt.%) cell exhibits a combination of a relatively large amount of dye adsorption, low transfer resistance, long electron lifetime, and IPCE, all possibly leading to enhanced *J*_sc _and *η *in DSSCs.

### Photovoltaic performance of composite TiO_2_/TNT DSSCs

**Figure 6:**
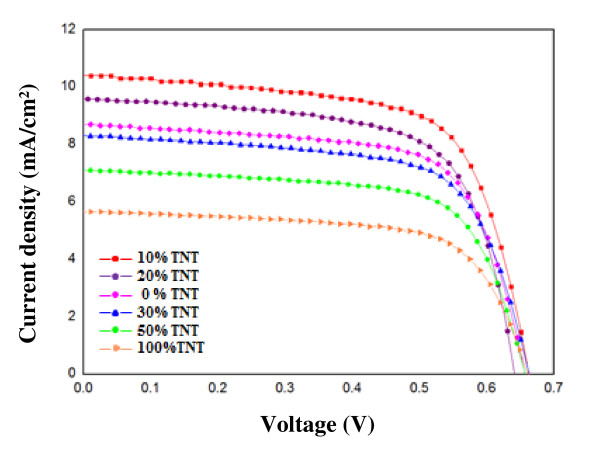
***I*-*V *characteristic of DSSC using hybrid TiO_2_/TNT films**.

Figure 6 shows the current-voltage photovoltaic performance curves of DSSCs based on the pure TiO_2 _cell, 10, 20, 30, 50, and 100 wt.% TNT cells, and cells without any TNT under AM 1.5 illumination (100 mW/cm^2^). One of the most important parameters of a solar cell is its photoelectric conversion efficiency, i.e., the ratio of the output power to the incident power. The energy conversion *η *can be estimated as:

$$\eta =\frac{V_{\text{oc}}\times J_{\text{sc}}\times \text{FF}}{P_{\text{s}}},$$

where *V*_oc _is the open-circuit voltage, *J*_sc _is the integral photocurrent density, FF is the fill factor:

$$\frac{V_{\max}\times J_{\max}}{V_{\text{oc}}\times J_{\text{sc}}},$$

and *P*_s _is the intensity of the incident light.

Table 1 summarizes the efficiency, fill factor, open-circuit voltage, and integral photocurrent for the corresponding solar cells. It can be seen that these DSSCs have a similar *V*_oc _of 0.65 V; because these flexible DSSCs have the same compositions, it makes sense that their *V*_oc _values are close. However, the *J*_sc _difference increases or decreases at various weight percentages of TNTs and TiO_2 _nanoparticles. A DSSC with a light-to-electric energy conversion efficiency of 4.57%, a short-circuit current density of 10.41 mA/cm^2^, an open-circuit voltage of 0.662 V, and a fill factor of 66.17% was achieved. For the hybrid 10 wt.% TiO_2_/TNT cell, the best results for conversion efficiency were obtained; the 100 wt.% TNT cell showed the worst results for conversion efficiency because the TNTs were in a random arrangement. The DSSCs based on TiO_2 _nanoparticle/TNT hybrids ranging from 0 to 100 wt.% showed higher values of FF, *V*_oc_, and *J*_sc_, and therefore higher efficiencies *η *than the cell based on pure TiO_2 _nanoparticles. It is obvious that the voltage of the DSSC with 10 wt.% TNTs is higher than that without TNTs.

**Table 1:** *J*_sc_, *V*_oc_, FF, and efficiency

	*V*_oc _(V)	*J*_sc _(mA/cm^2^)	FF (%)	*η *(%)
TiO_2 _nanoparticles	0.65	8.67	67.51	3.84
10 wt.% TNT	0.66	10.41	66.17	4.57
20 wt.% TNT	0.64	9.56	66.33	4.07
30 wt.% TNT	0.66	8.30	66.36	3.65
50 wt.% TNT	0.65	7.09	67.65	3.15
100 wt.% TNT	0.66	5.65	66.59	2.49

*V*_oc_, open-circuit voltage; *J*_sc_, integral photocurrent density; FF is the fill factor; *η*, energy conversion; TNT, TiO_2 _nanotube.

## Conclusions

DSSCs were constructed with TiO_2 _films made of different weight percentages of TNTs and TiO_2 _nanoparticles. The anatase-phase crystal property was found to be at its best at a hydrothermal temperature of 150°C for 12 h. The size and structure of the TNTs were adjusted by varying the hydrothermal temperature. It was found that the conversion efficiency of the DSSCs was highly affected by the properties of the TNTs. A DSSC with a light-to-electric energy conversion efficiency of 4.56% was achieved under a simulated solar light irradiation of 100 mW/cm^2 ^(AM 1.5). The DSSC based on a TiO_2_/TNT combination at the optimal weight percentage (10 wt.% TNT) showed better photovoltaic performance than the cell made purely of TiO_2 _nanoparticles.

## Competing interests

The authors declare that they have no competing interests.

## Authors' contributions

HWC and CHL presided over and fully participated in all of the work. HWC and CHL conceived and designed the experiments. CHL and SWR wrote the paper. All authors read and approved the final manuscript.
